# Crayfish plague in Japan: A real threat to the endemic *Cambaroides japonicus*

**DOI:** 10.1371/journal.pone.0195353

**Published:** 2018-04-04

**Authors:** Laura Martín-Torrijos, Tadashi Kawai, Jenny Makkonen, Japo Jussila, Harri Kokko, Javier Diéguez-Uribeondo

**Affiliations:** 1 Department of Mycology, Real Jardín Botánico (RJB-CSIC), Madrid, Spain; 2 Wakanai Fisheries Research Institute, Hokkaido, Japan; 3 Department of Environmental and Biological Sciences, University of Eastern Finland, Kuopio, Suomi-Finland; Uppsala Universitet, SWEDEN

## Abstract

Global introductions of aquatic species and their associated pathogens are threatening worldwide biodiversity. The introduction of two North American crayfish species, *Procambarus clarkii* and *Pacifastacus leniusculus*, into Japan in 1927 seems to have negatively affected native Japanese crayfish populations of *Cambaroides japonicus*. Several studies have shown the decline of these native populations due to competition, predation and habitat colonization by the two invasive North American crayfish species. Here, we identify an additional factor contributing to this decline. We report the first crayfish plague outbreaks in *C*. *japonicus* populations in Japan, which were diagnosed using both histological and molecular approaches (analyses of the internal transcribed spacer region). Subsequent analyses of the mitochondrial ribosomal rnnS and rnnL regions of diseased specimens indicate that these outbreaks originated from a *P*. *clarkii* population and identify a novel haplotype of *Aphanomyces astaci*, d3-haplotype, hosted by *P*. *clarkii*. Overall, our findings demonstrate the first two cases of crayfish plague in Japan, and the first case in a non-European native crayfish species, which originated from the red swamp crayfish *P*. *clarkii*. This finding is a matter of concern for the conservation of the native freshwater species of Japan and also highlights the risk of introducing crayfish carrier species into biogeographic regions harboring species susceptible to the crayfish plague.

## Introduction

Global movements of aquatic animals have facilitated the emergence of infectious diseases and have caused great losses in aquaculture and aquatic wildlife populations [1]. These movements often involve unintentional introductions that result in the establishment and spread of incidental “hitchhiking” species [2, 3]. For instance, several pathogens are known to have crept into new geographic areas and infected new hosts, resulting in emerging infectious diseases [1]. This is the case of *Aphanomyces astaci* Schikora 1903 (Oomycota), the pathogen responsible for the crayfish plague disease that caused the decimation and near extinction of the native European crayfish populations [4, 5]. This organism chronically infects its natural hosts, North American freshwater crayfish species [6], by establishing a balanced host-pathogen interaction [4]. However, the pathogen can easily kill susceptible species, *e*.*g*., native Australasian, European, Madagascan, and South American freshwater crayfish species.

The first crayfish plague outbreak was recorded in Europe in the 19^th^ century and coincided with the first introductions of non-native freshwater species, including the crayfish, into Europe [7]. Importation of freshwater species from North America continues to this day as a result of commercial trade in industries such as aquaculture, sport fishing, and the aquarium pet trade [3, 8]. Large-scale imports of North American crayfish species, and their spread by illegal translocations, have resulted in new crayfish plague outbreaks throughout Europe [9, 10] including Sweden, Finland, Spain, the United Kingdom, and Ireland [10–15]. As a consequence of its rapid spread and devastating effects, this pathogen is now listed among the 100 World’s Worst Invasive Alien Species, largely due to the worldwide distribution of the North American *A*. *astaci* carriers [16].

Various techniques, such as RAPD-PCR, AFLP and microsatellites, have been used to track the origin of *A*. *astaci* outbreaks [9, 17–20]. Recently, a new method that identifies mitochondrial ribosomal small (rnnS) and large (rnnL) subunit haplotypes of *A*. *astaci* from infected samples has also been successfully used to track this pathogen [21]. Thus far, five *A*. *astaci* haplotypes have been identified: a, b, d1, d2 and e. Each haplotype corresponds to one of the genetic groups identified by RAPD-PCR, *i*.*e*., groups A, B, C, D and E, with the exception of the a-haplotype, which is found in strains comprising RAPD-PCR groups A and C. These genetic groups and haplotypes can be tracked and associated with a particular North American crayfish species: RAPD-PCR groups B and C are linked to *P*. *leniusculus*, group D to *P*. *clarkii*, and group E to *Orconectes limosus*, besides genotype RAPD-PCR group A, which is likely the strain that caused the first known crayfish plague outbreaks in Europe and its original host is unknown [9, 17, 18].

Crayfish plague outbreaks have not yet been reported in some biogeographical regions identified as "hot spots" of crayfish biodiversity, *e*.*g*., Australia, Madagascar or South America [22–27] in spite of the presence of *A*. *astaci*-carriers, such as *P*. *clarkii*, in some regions of South America, including Argentina, Brazil, Colombia and Ecuador [28, 29]. Other biogeographical regions of key importance for crayfish biodiversity, such as Japan, which possesses an endemic crayfish species, *Cambaroides japonicus* De Haan 1841, are also threatened by invasive North American crayfish species. Prior to the 1920s, this species was widespread throughout Hokkaido Island [30]. However, its natural populations have drastically declined since the 1970s. Its current distribution range is restricted to Hokkaido and Aomori prefectures and to the northern parts of Akita and Iwate prefectures [31]. As a result, the Japanese Fisheries Agency in 1998 and the Environmental Agency in 2000 listed this species as endangered [32].

The North American crayfish species *P*. *clarkii* and *P*. *leniusculus* were introduced into Japan between 1927 and 1930 [26, 33]. These two invasive species have been implicated in the decline and disappearance of *C*. *japonicus* populations [34], specifically due to natural habitat colonization [26, 35], interspecific competition [30, 36], and predation [37]. However, few studies have focused on the pathogen *A*. *astaci*, which is chronically carried by *P*. *clarkii* and *P*. *leniusculus*, and its effects on this native species. Recently, several *P*. *clarkii* and *P*. *leniusculus* invasive populations in Japan have tested positive for the presence of *A*. *astaci* [38]. Although *C*. *japonicus* has been proved to be susceptible to *A*. *astaci* [4], no crayfish plague outbreaks have yet been reported in this native species [30].

In 2014 and 2015, two *C*. *japonicus* mass mortality events were observed in Sapporo, Hokkaido. Conservationists in Sapporo suspect these events may be due to the crayfish plague, given the proximity of some populations of *P*. *clarkii* to those of *C*. *japonicus*. Therefore, the main objective of this study is to determine whether these mortality events were caused by the crayfish plague pathogen *A*. *astaci*.

## Material and methods

### Ethical statement

All experimental procedures and animal manipulations, as well as field sampling, were performed according to the Japanese, EU and Spanish legislation. All analyses were carried out according to the regulations of Spanish Ministry MINECO. No additional permits were required for the laboratory studies, since the ethics approval in the Spanish law is not required for working with arthropod invertebrates. Moreover, this study was carried out in strict accordance with the recommendations and the protocols established in previous studies.

### Crayfish sampling

**Fig 1:**
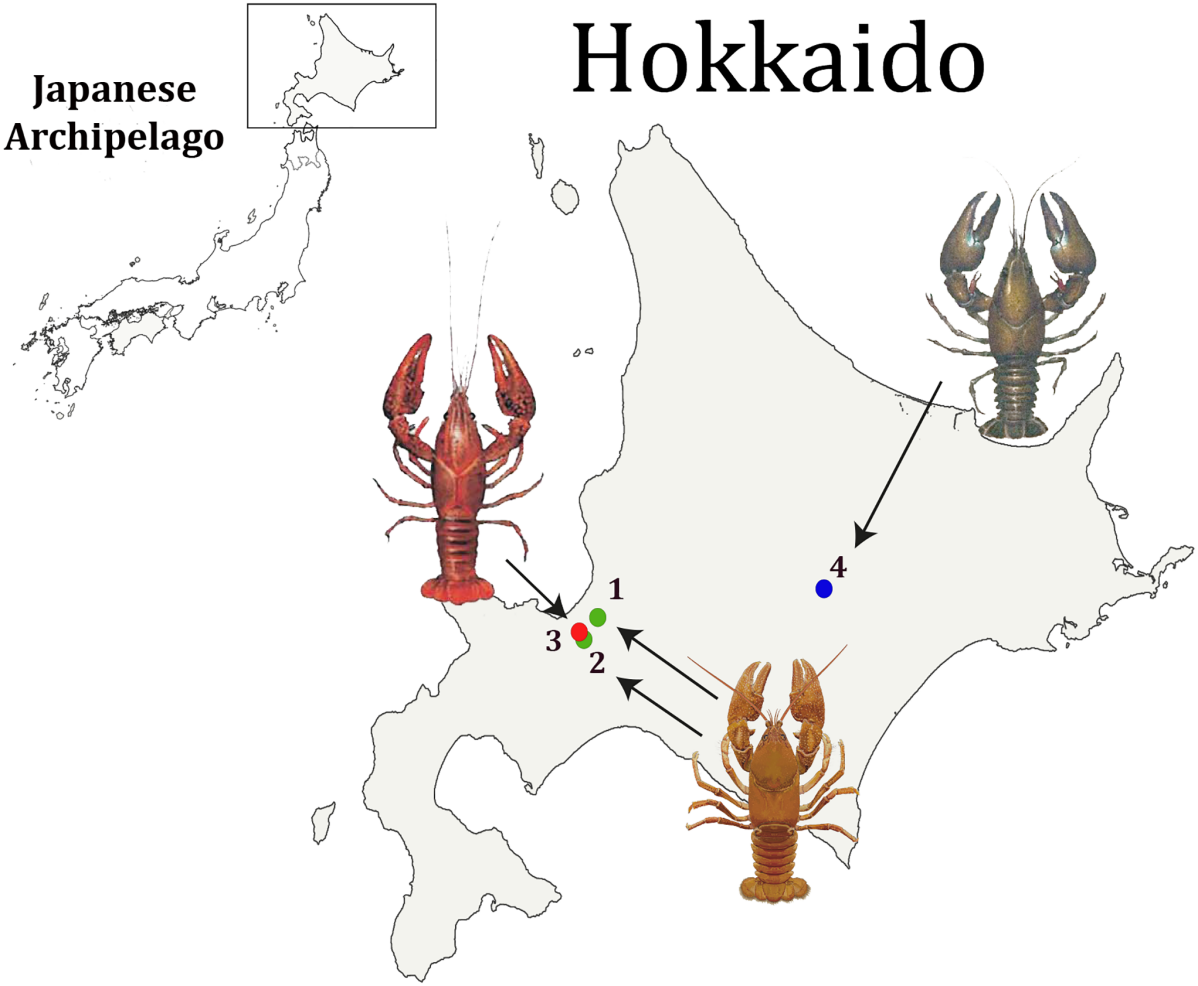
Location of the analyzed crayfish species across Hokkaido. Map indicating the locations of the *Cambaroides japonicus* populations that experienced mass mortality and the established populations of introduced species, *Procambarus clarkii* and *Pacifastacus leniusculus*, on Hokkaido Island (Japan). The green circles indicate the two suspected crayfish plague outbreak localities: **(1)** corresponds to Minami-ku and **(2)** corresponds to Ishikari River, both in Sapporo. The red circle **(3)** indicates the locality of the established *P*. *clarkii* population in Yasuharu (Sapporo), and the blue circle **(4)** the established *P*. *leniusculus* population in Lake Shikaribetsu.

A total of 15 dead specimens of *C*. *japonicus* originating from two mass mortality events were analyzed. The first mass mortality event occurred in Minami-ku, a ward south of the city of Sapporo, during October 2014. The second event occurred in Ishikari River during September 2015. Two *C*. *japonicus* individuals from the first event and four from the second were collected and preserved in ethanol 95% for further analyses (Fig 1, Table 1). Additionally, nine *C*. *japonicus* specimens from locations either nearby or far from the second mass mortality location were collected and analyzed (Table 1). All specimens were analyzed at the Laboratory of Molecular Systematics at the Real Jardín Botánico-CSIC, Madrid, Spain.

**Table 1:** Location and identification number of the analyzed crayfish species of Hokkaido. Location, date and *A*. *astaci* haplotype (if present) of the native *Cambaroides japonicus* and introduced *Procambarus clarkii* and *Pacifastacus leniusculus* specimens analyzed.

Species	Id	Location	Date	Incident	Extraction code	Haplotype
*C*. *japonicus*	CJ1	Minami-ku, Sapporo City.	October 2014	First mass mortality event detected	CE15/05-10	d3
	CJ2	Minami-ku, Sapporo City.			CE15/05-11	-----
*C*. *japonicus*	JPN-C1	Sapporo City	September 2015	Different station from the second mass mortality event	CE15/36-1	-----
	JPN-C2	Sapporo City			CE15/36-2	-----
	JPN-C3	Sapporo City			CE15/36-3	-----
*C*. *japonicus*	JPN-D1	Sapporo City	September 2015	Location next to the second mass mortality	CE15/36-4	-----
	JPN-D2	Sapporo City			CE15/36-5	-----
	JPN-D3	Sapporo City			CE15/36-6	-----
	JPN-D4	Sapporo City			CE15/36-7	-----
	JPN-D5	Sapporo City			CE15/36-8	d1
	JPN-D6	Sapporo City			CE15/36-9	-----
*C*. *japonicus*	JPN-A1	Sapporo City	September 2015	Second mass mortality event detected	CE15/36-10	d1
	JPN-A2	Sapporo City			CE15/36-11	-----
	JPN-A3	Sapporo City			CE15/36-12	-----
	JPN-A4	Sapporo City			CE15/36-13	-----
*P*. *leniusculus*	JPN-P1	Lake Shikaribetsu	October 2015	Established population	CE15/35-1	b
	JPN-P2	Lake Shikaribetsu			CE15/35-2	-----
	JPN-P3	Lake Shikaribetsu			CE15/35-3	-----
	JPN-P4	Lake Shikaribetsu			CE15/35-4	-----
	JPN-P5	Lake Shikaribetsu			CE15/35-5	-----
	JPN-P6	Lake Shikaribetsu			CE15/35-6	-----
*P*. *clarkii*	JPN-P7	Yasuharu	October 2015	Established population	CE15/35-7	-----
	JPN-P8	Yasuharu			CE15/35-8	-----
	JPN-P9	Yasuharu			CE15/35-9	-----
	JPN-P10	Yasuharu			CE15/35-10	d3
	JPN-P11	Yasuharu			CE15/35-11	d3
	JPN-P12	Yasuharu			CE15/35-12	d3
	JPN-P13	Yasuharu			CE15/35-13	d3

To test the prevalence of the pathogen *A*. *astaci* in introduced North American species, *P*. *clarkii* specimens from a population inhabiting Yasuharu, a vicinity with known *C*. *japonicus* populations (Fig 1) and *P*. *leniusculus* specimens from an established population in Shikaribetsu Lake in a central region of eastern Hokkaido Island (Fig 1) were collected during October 2015 for further analysis (Table 1).

### Macroscopic and microscopic examination

All analyzed crayfish were examined macroscopically to check for the presence of melanized areas and microscopically for the presence of hyphae in the soft cuticle, both of which are indicators of *A*. *astaci* infection. For microscopic examination, the subabdominal cuticle was removed and observed using an Olympus CKX41SF inverted microscope (Olympus Optical, Tokyo, Japan). Light micrographs of the colonizing hyphae were captured using a QImaging Micropublisher 5.0 digital camera (QImaging, Burnaby, BC, Canada). Digital image analysis was performed using the software Syncroscopy-Automontage (Microbiology International Inc., Frederick, MD) as described by Diéguez-Uribeondo et al. 2003 [39].

### Molecular analyses

#### Genomic isolation, PCR amplification and sequencing

Subabdominal soft cuticle samples were rehydrated from ethanol into TE buffer (TRIS 10 mM/ EDTA 1 mM, pH 8). Each sample was rinsed three times for 1 hour with TE prior to an overnight wash. Samples were transferred into individual 2 ml Eppendorf tubes, frozen at -80 °C and then lyophilized in a freeze dryer VirTis BenchTop K for 24 hours (≤-50 °C; ≤ 20 mTorr). The samples were then mechanical ruptured using a TissueLyser (QIAGEN, Venlo, The Netherlands). Genomic DNA was isolated with the E.Z.N.A.^®^ Insect DNA Kit (Omega Bio-Tek, Norcross, Georgia, USA). The extracted DNA and *A*. *astaci* diagnostic primers 42 [40] and 640 [41] (which amplify the ITS1 and ITS2 surrounding the 5.8S rDNA, and anchored in ITS1 and ITS2 regions, respectively) were used for a single round of PCR according to the assay described by Oidtmann et al. 2006 [40]. As a positive control, DNA extracted from a pure culture of the *A*. *astaci* strain AP03 [42], was used; distilled Milli-Q water was used as a negative control. Amplified products were analyzed by electrophoresis in 1% agarose TAE gels stained with SBYR^®^ Safe (Thermo Fisher Scientific, Waltham, MA, USA). Both strands of PCR amplified products were sequenced using an automated sequencer (Applied Biosystems 3730xl DNA, Macrogen, The Netherlands). Each sequence strand was assembled and edited with Geneious^®^ 10.0.2 [43]. BLAST searches were performed to verify the identities of the obtained sequences.

#### Phylogenetic and haplotype analyses

Specimens of *C*. *japonicus*, *P*. *clarkii* and *P*. *leniusculus* that tested positive for *A*. *astaci* based on diagnostic primers 42 [40] and 640 [41] were further analyzed to characterize the phylogenetic relationships and haplotypes of *A*. *astaci* present in the crayfish cuticles. Mitochondrial rnnS and rnnL sequences were obtained as described by Makkonen et al. [21]. Briefly, mitochondrial ribosomal rnnS and rnnL primers pairs (AphSSUF/AphSSUR and AphLSUF/AphLSUR, respectively) [21] were used for the pathogen characterization. The aforementioned positive and negative controls were also included. Amplified products were analyzed and sequenced as described above. However, in this case, amplified products were first purified using a QIAquick PCR Purification Kit (QIAGEN).

Sequences were assembled and edited using the program Geneious^®^ 10.0.2 [43] and two phylogenetic approximations, Bayesian Interference (BI) and Maximum Likelihood (ML), were employed to reconstruct phylogenetic relationships as described by Makkonen et al. [21]. The following haplotype sequences from GenBank were used as references in the approximations: accession numbers MF973121–MF973149 for rnnS and MF975950–MF975978 for rnnL. *Aphanomyces frigidophilus* was used as outgroup. We analyzed rnnS and rnnL independently, and a concatenated rnnS and rnnL dataset with the same parameters.

## Results

### Macroscopic and microscopic examination

**Fig 2:**
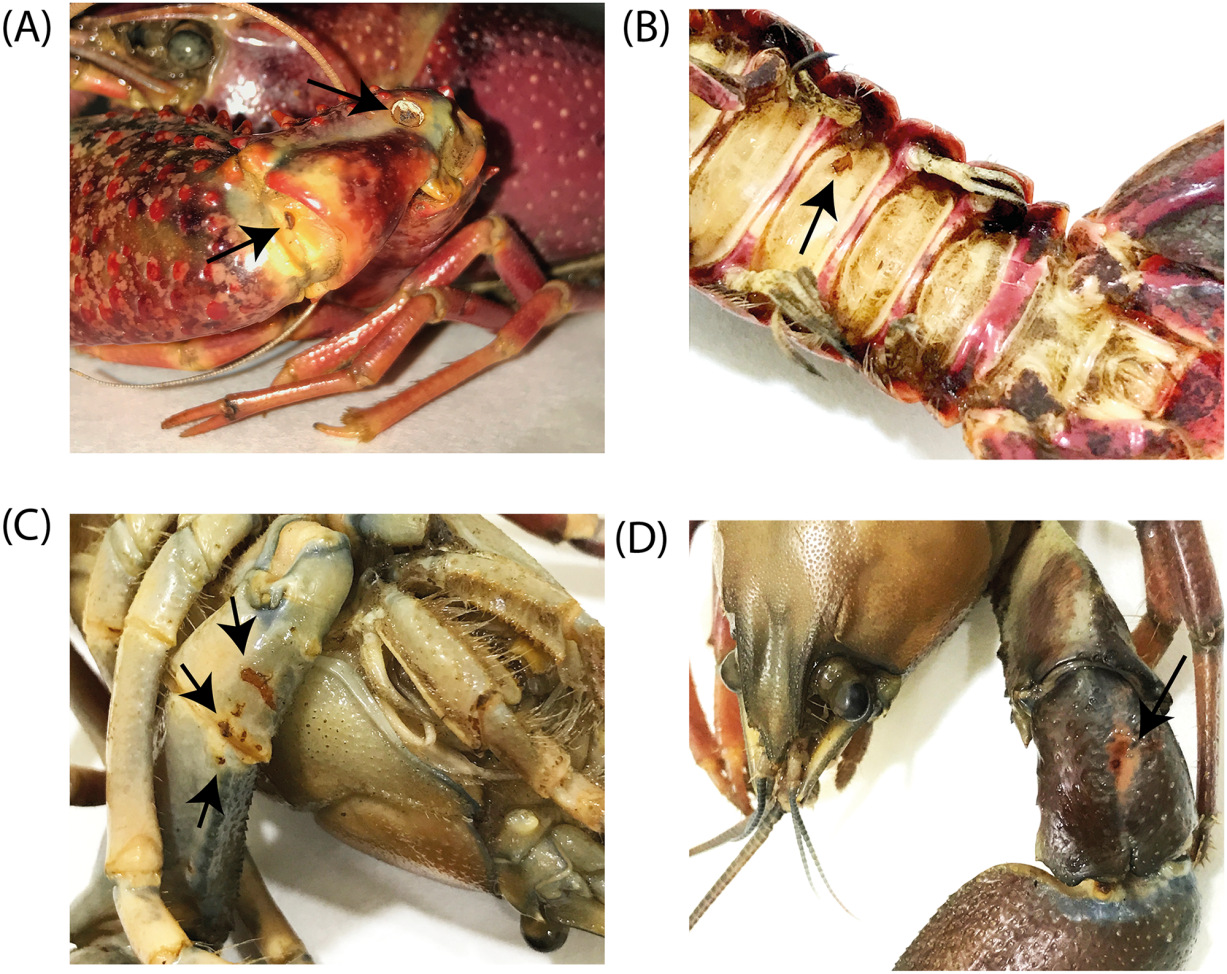
*Aphanomyces astaci* colonization and immune reaction in North American crayfish. North American crayfish species showing immune responses to *Aphanomyces astaci* infection. Photographs of (A, B) *P*. *clarkii* and (C, D) *P*. *leniusculus* specimens. Melanin formation, visualized as melanized patches (arrows), characterizes a strong immune response against *A*. *astaci* infections on the **(A)** joints of a chela and **(B)** subabdominal cuticle of a *P*. *clarkii* specimen, and on the **(C)** ventral and **(D)** dorsal chela surfaces of a *P*. *leniusculus* specimen.

**Fig 3:**
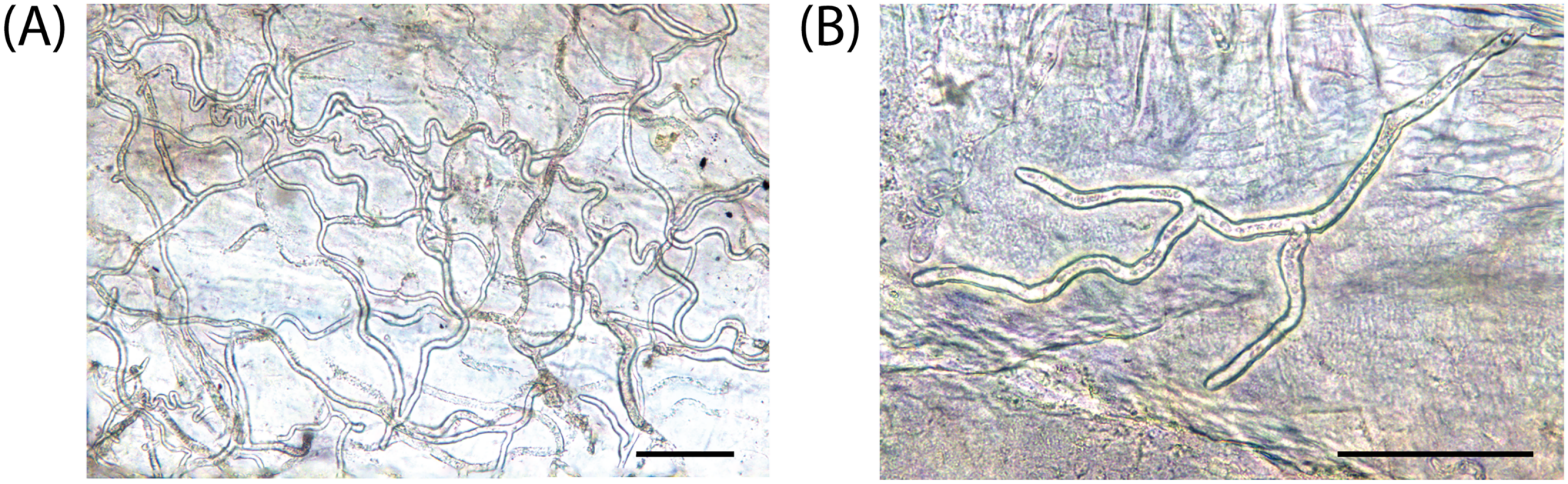
Hyphal colonization of the cuticle of susceptible *Cambaroides japonicus*. Montage micrographs of hyphae growing within the cuticle. **(A)** Cuticle overgrown by hyphae; **(B)** Single hypha. Bar = 100 μm. DNA extractions from these pieces of subabdominal cuticle tested positive for *A*. *astaci* diagnostic primers 42 and 640 specific primers and haplotyping mitochondrial ribosomal primers rnnS and rnnL primers pairs.

Macroscopic observations showed that all *P*. *clarkii* and *P*. *leniusculus* specimens exhibited characteristic melanized areas on the subabdominal cuticle, joints and chelae (Fig 2). Melanized patches or spots on the *C*. *japonicus* cuticles were not observed. However, microscopic examination of the subabdominal soft cuticle of the *C*. *japonicus* samples revealed an abundance of non-melanized *A*. *astaci* hyphae (Fig 3). These hyphae had rounded tips and similar diameters, ca 10 μm, characteristics of an *A*. *astaci* infection. However, no melanized hyphae or micro-melanized spots were detected in any of the *C*. *japonicus* samples analyzed (Fig 3).

### Molecular analyses

One *C*. *japonicus* from each of the two mortality event localities, one *C*. *japonicus* from the location proximate to the second outbreak, one *P*. *leniusculus* and five *P*. *clarkii* tested positive for *A*. *astaci* based on amplification of the ITS region with the diagnostic primers 42 [40] and 640 [41] (Table 1). BLAST analyses of the sequenced PCR products showed 100% similarity to strain SAP0877 *Aphanomyces astaci* (GenBank accession number KX555484), which originated from *P*. *clarkii* [44].

**Fig 4:**
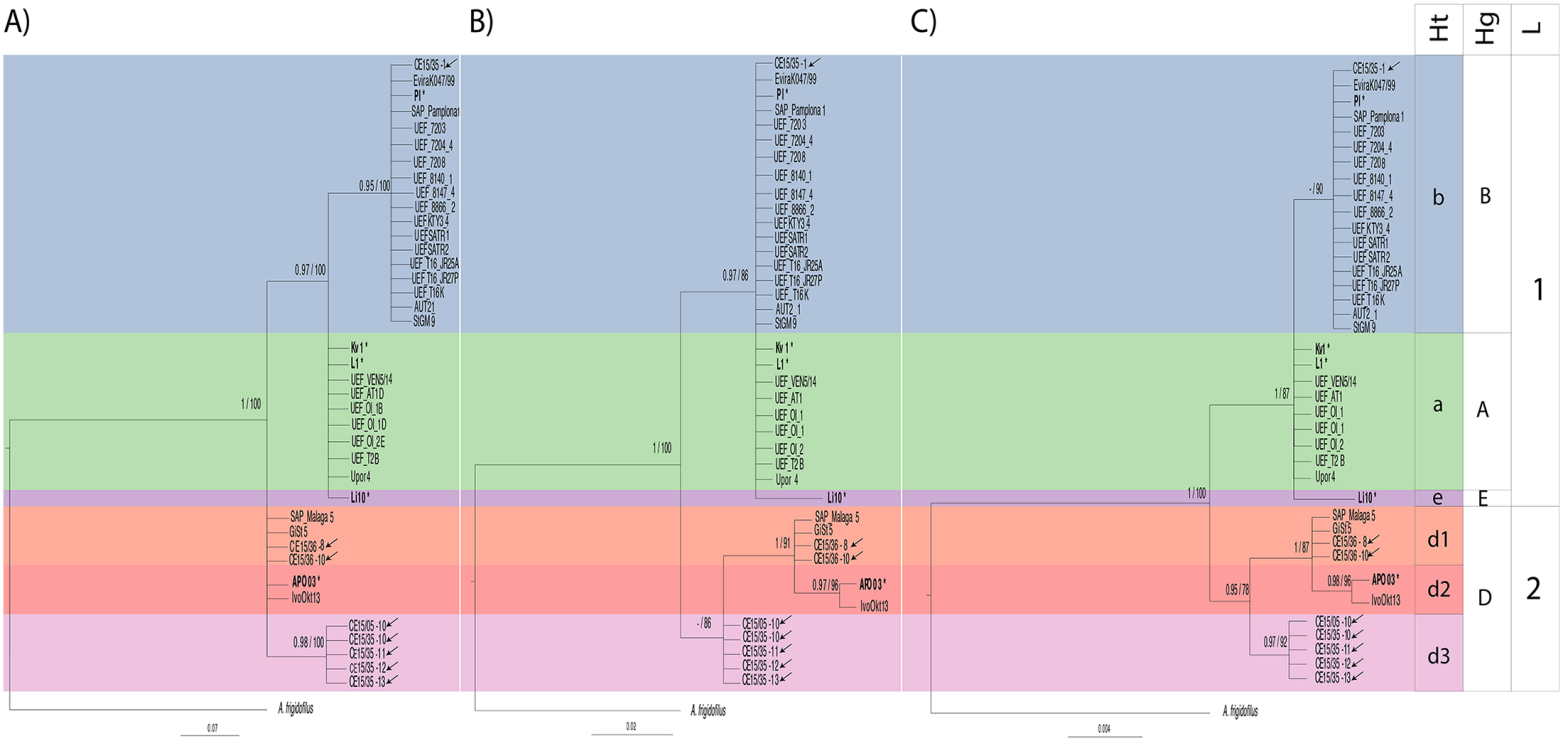
Phylogenetic analyses of *A*. *astaci* mitochondrial regions. Bayesian phylogenetic analyses of *A*. *astaci* mitochondrial rnnS, rnnL and concatenated rnnS + rnnL sequences obtained from infected crayfish specimens (arrows) of the native *C*. *japonicus* and the invasive *P*. *clarkii* and *P*. *leniusculus* found on the island of Hokkaido, Japan. **(A)** Bayesian phylogenetic tree based on the rnnS sequences. **(B)** Bayesian phylogenetic tree based on the rnnL sequences. **(C)** Bayesian phylogenetic tree based on the concatenated rnnS + rnnL sequences. Values above the branches represent the Bayesian posterior probabilities (>0.95) and ML bootstrap support values (> 75), respectively. Scales bar for phylogenetic analysis indicates substitutions per site. The original strains used as references and identified in previous studies by RAPD-PCR [9, 17, 18] are indicated in bold and with a star key (*) correspond to group A (L1), group B (Pl), group C (Kv1), group D (AP03) and group E (Li10). Abbreviations: Ht, haplotypes; Hp, haplogroups; L, lineages.

PCR amplification of the mitochondrial ribosomal rnnS and rnnL regions of the infected specimens produced 476 base pairs (bp) and 355 bp fragments, respectively (GenBank accession number for rnnS MG905008- MG905015 and for rnnL MG905000- MG905007). The BI and ML analyses of the rnnS (Fig 4A) and rnnL (Fig 4B) regions recovered congruent topologies and indicated the presence of a novel haplotype, d3. Analysis of the concatenated rnnS and rnnL dataset supported a new clade comprised of the novel d3-haplotype, which corresponds to the D-haplogroup (Fig 4C). One of the *C*. *japonicus* specimen from the first crayfish plague outbreak and five of the *P*. *clarkii* specimens showed this haplotype (Table 1, Fig 4C). The presence of the d1-haplotype, grouped within the D-haplogroup, was supported for one of the *C*. *japonicus* specimen from the second crayfish plague outbreak (and one specimen from the proximate locality). The infected *P*. *leniusculus* specimen from Shikaribetsu Lake grouped within the b-haplotype in the B-haplogroup (Table 1, Fig 4C).

**Fig 5:**
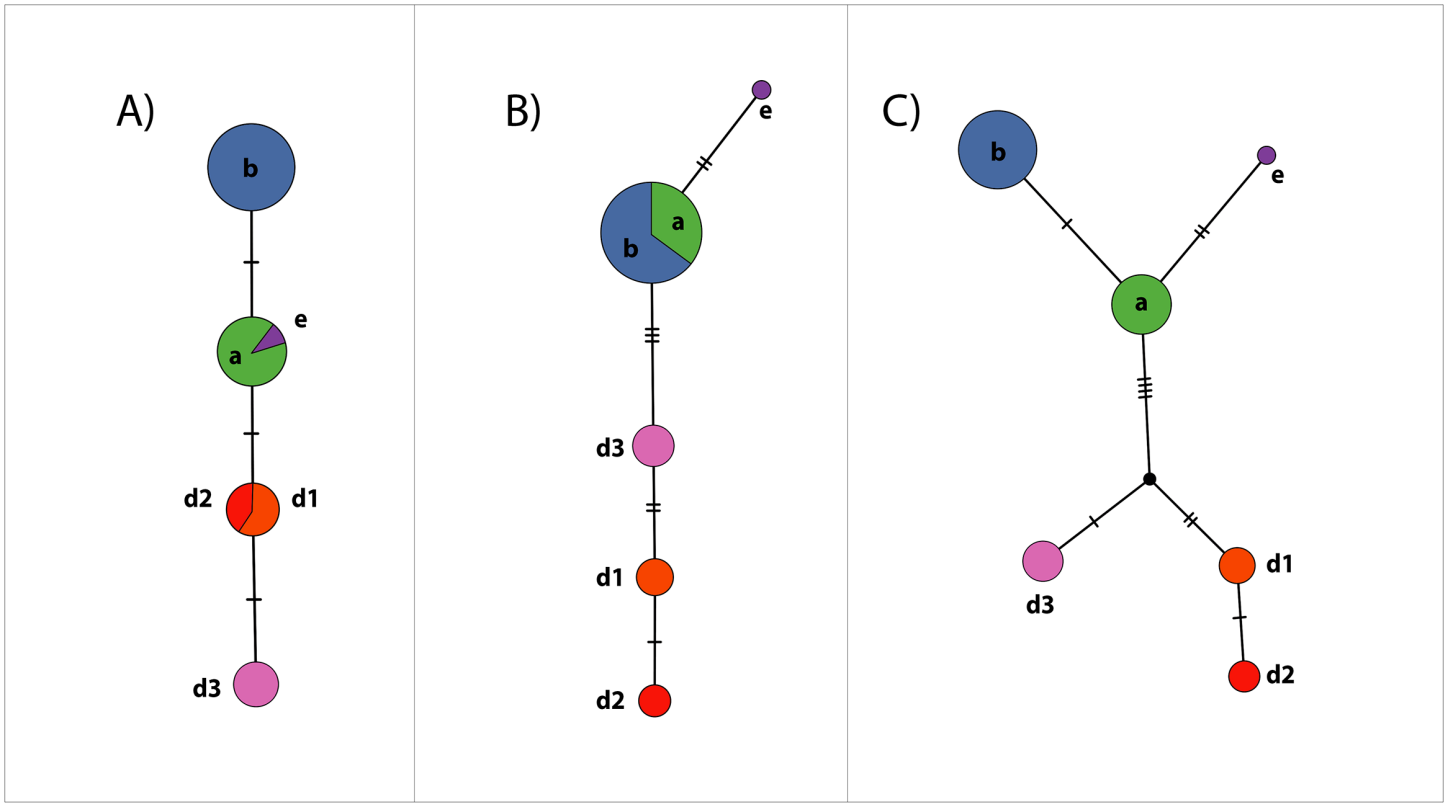
Haplotype network analyses of *A*. *astaci* mitochondrial regions. Haplotype network analyses of the *A*. *astaci* mitochondrial rnnS, rnnL, and concatenated rnnS + rnnL sequences obtained from the infected specimens analyzed in this study, generated by statistical parsimony. The area of the circles is proportional to the number of sequences. **(A)** Haplotype network based on the rnnS sequences. **(B)** Haplotype network based on the rnnL sequences. **(C)** Haplotype network based on the concatenated rnnS + rnnL sequences. Mutation steps between haplotypes are shown as hatch marks.

Observed haplotype diversity (Fig 5) is consistent with the phylogenetic analyses (Fig 4). The amplicons corresponding to the rnnS region registered three segregating sites, resulting in four different haplotypes (Fig 5A) (Table 2), whereas the amplicons from the rnnL region registered eight segregating sites and five different haplotypes (Fig 5B) (Table 2). The concatenated rnnS + rnnL dataset showed a total of 11 segregating sites, supporting the existence of six haplotypes (Fig 5C) (Table 2).

**Table 2:** DNA sequence polymorphisms and haplotypes found in *Aphanomyces astaci* sequences for the mitochondrial ribosomal rnnS and rnnL subunits. DNA sequence polymorphisms detected in rnnS and rnnL subunits for 6 haplotypes. The second line shows the relative position in the concatenated sequence (rnnS + rnnL) of 831 pb. A dash (–) denotes a single nucleotide indel.

	rnnS			rnnL								
	148	367	397	510	534	546	616	625	627	655	805	813
**a-haplotype**	T	**A**	G	G	T	G	-	**A**	C	**C**	G	**A**
**b-haplotype**	T	G	G	G	**A**	**A**	**T**	T	**A**	T	**A**	C
**d1-haplotype**	**C**	**A**	G	G	T	G	-	**A**	C	T	G	C
**d2-haplotype**	T	G	**A**	G	**A**	G	-	T	**A**	T	G	C
**d3-haplotype**	T	G	G	**A**	**A**	**A**	-	T	**A**	T	**A**	C
**e-haplotype**	T	**A**	G	G	T	G	**T**	**A**	C	T	G	C

## Discussion

In this study, we report and describe the first cases of crayfish plague mass mortalities in Japan using histological and molecular approaches. These two cases also represent the first reported crayfish plague outbreaks in a native crayfish population outside of Europe and Asia minor. We found that these mass mortalities in *C*. *japonicus* populations originated from *P*. *clarkii* populations, based on the presence of the *A*. *astaci* d1- and d3-haplotypes. The d3-haplotype is a novel haplotype reported here for the first time. These two haplotypes belong to the D-haplogroup, which is associated with *P*. *clarkii*. Furthermore, we detected the novel d3-haplotype in *P*. *clarkii* specimens from Japan.

Although the susceptibility of *C*. *japonicus* species to *A*. *astaci* was first demonstrated by Unestam in 1969 [4], no massive mortalities associated with *A*. *astaci* have been described until our study. We have shown that the pathogen *A*. *astaci* can cause mass mortalities among native Japanese crayfish populations as it has in native European crayfish populations [5]. Furthermore, histological analyses of *C*. *japonicus* tissues revealed abundant and non-melanized hyphae of *A*. *astaci* growing within the cuticle, similar to what has been observed in European species [45]. In contrast to the highly resistant North American crayfish species, *P*. *clarkii* and *P*. *leniusculus* [45, 46], we did not observed signs of resistance against this pathogen, *i*.*e*., melanized hyphae or spots, in *C*. *japonicus*. The North American species are often chronically infected by the pathogen due to a strong immune response [47], which contains the pathogen but allows the dispersion of its infectious units, the biflagellate zoospores, which can then colonize new crayfish hosts, such as *C*. *japonicus*.

The crayfish plague outbreaks in Minami-ku and Ishikari River occurred in the vicinity of a *P*. *clarkii* population in Yasuharu (Fig 1). In this study, we also provide evidence, based on mtDNA rnnS and rnnL analyses of clinical samples, that both outbreaks are consequences of the transmission of the pathogen from *P*. *clarkii*. Our analyses indicated that the *A*. *astaci* haplotype present in *C*. *japonicus* from Minami-ku and *P*. *clarkii* from Yasuharu is the d3-haplotype. On the other hand, the specimens from the second mass mortality event in Ishikari River presented the d1-haplotype (of the D-haplogroup). This finding suggests that a different *P*. *clarkii* population infected these particular *C*. *japonicas* specimens. These results represent an additional concern in Japan, as the two haplotypes associated with the crayfish plague outbreaks here belong to a virulent D-haplogroup. The physiological properties of this haplogroup’s strains allow them to grow, sporulate, and produce zoospores at higher temperatures than other strains [17]. Although the two *P*. *clarkii* associated haplotypes, d1- and d3-haplotypes, were found to be the cause of the mass mortalities, we also detected the presence of the b-haplotype in its natural carrier *P*. *leniusculus* from Shikaribetsu Lake in the central region of eastern Hokkaido Island. Therefore, two strains with different temperature preferences are now in Japan, which creates the potential for native *C*. *japonicus* to be infected by the pathogen at a wider temperature range. This is also very similar to the scenario in Southern Europe, where both B- and D-haplogroups (with their respective b- and d1- and d2-haplotypes) coexist and have driven the native European crayfish species *Austropotamobius pallipes* to a risk of extinction [10, 13, 48].

Numerous studies have warned about the risks concerning the North American crayfish carrying *A*. *astaci* [9, 46, 49–54]; these risks were specifically discussed for Japan by Mrugala in 2016 [38]. Several studies carried out in Japan have indicated that aggressive interaction for shelter and predation by *P*. *leniusculus* is causing the decline of *C*. *japonicus* [30, 37, 55]. However, *P*. *clarkii* has not been implicated in its decline, until now. The risk posed by *P*. *clarkii* was probably overlooked as *C*. *japonicus* and *P*. *clarkii*, generally speaking, have different habitats due to their individual environmental requirements [32]. Thus, it should be taken into account that *P*. *clarkii* possesses great adaptability, making it a successful colonizer in the aquatic ecosystem of Japan [56], including in *C*. *japonicus* habitats.

Our results demonstrate that the pathogen *A*. *astaci* constitutes an actual threat to the endemic and endangered *C*. *japonicus*. Consequently, we urge authorities to rapidly develop and implement action plans, including strategies that aim to restore and manage native *C*. *japonicus* populations and to control and/or eradicate invasive crayfish species, especially *P*. *clarkii* and *P*. *leniusculus*. In Europe, the implementation of similar plans have allowed the conservation of the native European crayfish [57]. Moreover, preventing new introductions and translocations of North American crayfish species in Japan needs to be prioritized. The results presented in this study also pose as a warning of the potential risk of similar episodes of *A*. *astaci* spreading with alien crayfish to continents thus far free of the crayfish plague pathogen.
